# Transcriptional activation and localization of expression of *Brassica juncea *putative metal transport protein *BjMTP1*

**DOI:** 10.1186/1471-2229-7-32

**Published:** 2007-06-18

**Authors:** Balasubramaniam Muthukumar, Bakhtiyor Yakubov, David E Salt

**Affiliations:** 1 Department of Horticulture and Landscape Architecture, 625 Agricultural Mall Drive, Purdue University, West Lafayette, IN 47907-1392 USA

## Abstract

**Background:**

Metal hyperaccumulators, including various Thlaspi species, constitutively express the putative metal transporter *MTP1 *to high levels in shoots. Here we present data on the transcriptional regulation and localization of expression of the homologous gene *BjMTP1 *in *Brassica juncea*. Though *B. juncea *lacks the ability to hyperaccumulate metals, its relatively high biomass, rapid growth and relatedness to true metal hyperaccumulating plants makes it a promising starting point for the development of plants for phytoremediation. Our goal in this study is to determine the transcriptional regulation of *MTP1 *in order to start to better understanding the physiological role of MTP1 in *B. juncea*.

**Results:**

Steady-state mRNA levels of *BjMTP1 *were found to be enhanced 8.8, 5.9, and 1.6-fold in five-day-old *B. juncea *seedlings after exposure to Ni^2+^, Cd^2+ ^or Zn^2+^, respectively. This was also reflected in enhanced GUS activity in *B. juncea *seedlings transformed with *BjMTP1 promoter::GUSPlus *after exposure to these metals over a similar range of toxicities from mild to severe. However, no increase in GUS activity was observed after exposure of seedlings to cold or heat stress, NaCl or hydrogen peroxide. GUS expression in Ni^2+ ^treated seedlings was localized in roots, particularly in the root-shoot transition zone. In four- week- old transgenic plants *BjMTP1 *promoter activity also primarily increased in roots in response to Ni^2+ ^or Cd^2+ ^in plants transformed with either GUS or mRFP1 as reporter genes, and expression was localized to the secondary xylem parenchyma. In leaves, *BjMTP1 *promoter activity in response to Ni^2+ ^or Cd^2+ ^spiked after 24 h then decreased. In shoots GUS expression was prominently present in the vasculature of leaves, and floral parts.

**Conclusion:**

Our studies establish that a 983 bp DNA fragment upstream of the *BjMTP1 *translational start site is sufficient for the specific activation by Ni^2+ ^and Cd^2+ ^of *BjMTP1 *expression primarily in roots. Activation of expression by both metals in roots is primarily localized to the xylem parenchyma cells. This study is the first to identify specific Ni^2+ ^and Cd^2+ ^transcriptional regulation and tissue localization of *BjMTP1.*

## Background

Plant Cation Diffusion Facilitator (CDF) family members have been suggested to be involved in metal ion transport, and implicated in metal resistance in plants [1]. However, the physiological role of these transporters is not well understood. *AtMTP1 *(*ZAT*) from *Arabidopsis thaliana *was the first member of the CDF family to be characterized in plants [2]. When ectopically over expressed in *A. thaliana AtMTP1 *confers enhanced resistance to Zn^2+^, and increased Zn^2+ ^accumulation in roots [2]. Enhanced Zn^2+ ^resistance and accumulation was attributed to increased vacuolar sequestration of Zn^2+^. More recent evidence has established that AtMTP1 is predominantly localized at the tonoplast membrane in both root and shoot tissue in *A. thaliana *[3,4]. Consistent with a role in Zn^2+ ^transport into the vacuole, reduction in expression of *AtMTP1 *leads to increased sensitivity to Zn^2+^, but not Co^2+^, Cd^2+^, Ni^2+ ^or Mn^2+ ^[3,4]. Reduced expression of *AtMTP1 *also leads to decreased accumulation of Zn^2+ ^in shoots [4]. Reconstruction of AtMTP1 in proteoliposomes, and expression of the *AtMTP1 *cDNA in *Xenopus *oocytes has provided direct evidence that AtMTP1 is competent to transport Zn^2+ ^but not Cd^2+ ^or Co^2+ ^[4,5]. To date all studies have found that *AtMTP1 *expression is not modulated by exposure to elevated Zn^2+^, Cd^2+^, Co^2+^, Cu^2+^, Fe^2+ ^or Mn^2+ ^[2,4]. Expression of *AtMTP1 *in *A. thaliana *occurs throughout the plant, though transcript levels are higher in roots than shoots in young seedlings, and expression is higher in the inflorescences [4]. However, little is known about tissue-specific expression patterns of *AtMTP1*. Such information is critical if we are to integrate the currently available data into a model describing how MTP1 functions within the physiological context of the whole plant.

Homologues of *AtMTP1 *have been found in other plant species including the hyperaccumulators *Thlaspi goesingense *[6], *Thlaspi caerulescens *[7] and *Arabidopsis halleri *[8]. It has been suggested that the constitutively higher shoot expression of *MTP1 *in these hyperaccumulators is involved in metal hyperaccumulation [6,7], and recent genetic evidence supports this hypothesis [8]. However, the role of the MTP1 protein in hyperaccumulation is still unknown. In the Zn^2+ ^hyperaccumulator *A. halleri *AhMTP1 appears to be localized at the tonoplast membrane when transiently expressed as an AhMTP1::GFP fusion in *A. thaliana *protoplasts [8]. Similar localization has also been observed for an MTP1 homologue from poplar [9]. However, in a similar experiment with *TgMTP1 *from the Zn^2+^/Ni^2+ ^hyperaccumulator *T. goesingense *the GFP fusion protein was found to localize to the plasma membrane when transiently expressed in *A. thaliana *protoplasts derived from shoot tissue [10]. Resolution of this interesting difference awaits further comparative studies.

*Brassica juncea *is an amphidiploid plant, resulting from the hybridization of the crop Brassicas *Brassica nigra *and *Brassica campestris *(syn. *Rapa*; [11]). It contains the conserved genomes of both of its diploid parents [12], and is self compatible, unlike other crop *Brassica *species. Due to its rapid growth and large biomass *B. juncea *has been considered as a possible plant for use in the phytoextraction process, for the removal of pollutant metals from soils by their accumulation into harvestable above ground biomass [13]. The relatedness of *B. juncea *to numerous hyperaccumulators in the Brassicaceae family, and its ability to be self fertilized makes *B. juncea *a potentially good recipient organisms for the bioengineering of a practical phytoextraction plant using genetic material derived from natural hyperaccumulator species [14]. However, such promise has yet to be realized.

*Brassica juncea *have been shown to accumulate heavy metals, though *B. juncea *is not a hyperaccumulator [15-17]. However, there are no reports available that attribute a specific role of BjMTP1 in metal accumulation in *B. juncea*. Here we report that both Ni^2+ ^and Cd^2+ ^induce transcriptional activation of *BjMTP1 *in whole seedlings, as well as root and shoot tissue of mature plants, where as Zn^2+ ^has very little effect on *BjMTP1 *expression. We establish that a 983 bp DNA fragment upstream of the *BjMTP1 *translational start site is sufficient for this regulation, and that expression in roots is specifically localized to the xylem parenchyma cells. This study is the first to identify transcriptional regulation and tissue localization of *BjMTP1*. However, further work is needed to understand the functional role of BjMTP1 in *B. juncea's *response to Ni^2+ ^and Cd^2+^.

## Results

### *MTP1 *sequences from *B. juncea *and its parents

Using *AtMTP1 *primers *MTP1 *homologues were amplified from *B. juncea *and its two parents *B. nigra *and *B. campestris *(GenBank accession numbers EF128447, EF128446 and Ef128445, respectively). All Brassica sequences showed 80% similarity to *AtMTP1*, and all were devoid of introns similar to AtMTP1. *Brassica nigra *and *B. campestris MTP1 *showed 97% and 95% similarity to the *B. juncea MTP1 *sequence, respectively. A phylogenetic analysis of plant MTP1 sequences revealed that all Brassica MTP1 sequences fall into a monophyletic clade, with MTP1 from *B. juncea *being equally related to MTP1 from both its parents [see Additional file 1]. Isolation of the DNA sequence 5' of *BjMTP1 *(see below) revealed that the next gene upstream of *BjMTP1 *is 88% similar to the *A. thaliana *gene locus At2g46790. At2g46790 is the next upstream gene to *AtMTP1 *in *A. thaliana*. Such results show that synteny is conserved within the region of *MTP1 *between *B.juncea *and *A.thaliana*, and provides further strong evidence that the MTP sequence identified in *B. juncea *is the *B. juncea *homologue of *AtMTP1*.

### Isolation and characterization of *BjMTP1 *promoter

A two-step genome walking technique was used to isolate DNA 5' of the *B. juncea MTP1 *translational start site. As a first step a 1,561 bp PCR product was amplified from the DraI genomic DNA library and cloned. To isolate additional upstream sequence further genome walking was performed using the PvuII genomic DNA library giving a new 1,721 bp PCR product. Sequence analysis of this 1,721 bp DNA fragment indicated it contains not only the remaining sequence 5' of *BjMTP1 *but also partial sequence of the next gene upstream of *BjMTP1 *which showed 88% similarity to the *A. thaliana *gene locus At2g46790 (unknown protein). The 1,561 bp and 1,721 bp DNA fragments were compared and a contiguous 1,786 bp fragment determined. The complete 5' upstream region (1,786 bp), as well as the 1,561 bp fragment, were amplified from the *B. juncea *genomic DNA and the PCR products used for all further analysis. A 983 bp DNA fragment 5' of *BjMTP1 *translational start site was predicted to contain the majority of the regulatory elements within the complete 1,786 bp upstream region, and was therefore also amplified and used for further experiments.

### Transcriptional activation of *BjMTP1 *by various metal ions

**Figure 1:**
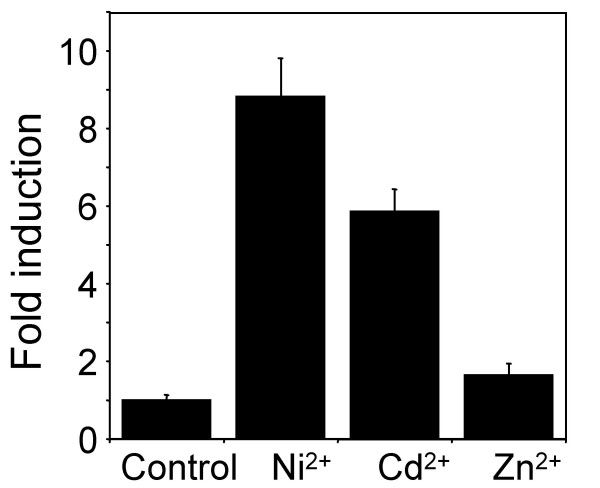
Metal regulation of steady-state levels of *BjMTP1 *mRNA. Steady-state levels of *BjMTP1 *mRNA in five-day-old dark grown *B. juncea *seedlings exposed to Ni^2+ ^(25 μM), Cd^2+ ^(5 μM) or Zn^2+ ^(75 μM) for 48 h measured by qRT-PCR. Data are presented as fold induction (2^^-^ΔΔC_T_), and represent the mean (± standard deviation) of three biological replicates each analyzed four time by qRT-PCR.

Total RNA was isolated from dark grown seven-day-old *B. juncea *seedlings which had been treated with either 5 μM Cd^2+^, 25 μM Ni^2+ ^or 75 μM Zn^2+ ^for 48 h, treatments that also produced maximal GUS activity in transgenic plants expressing a GUS report gene (see below). These three metals were chosen for this study based on the fact that the hyper accumulators that are known to have constitutively elevated MTP1 expression hyperaccumulator Cd, Ni or Zn. *BjMTP1 *mRNA were quantified using real time quantitative RT-PCR (qRT-PCR) and normalized to *BjACTIN2 *as an internal control. Steady-state levels of *BjMTP1 *mRNA were found to be increased after exposure of seedlings to Cd^2+ ^or Ni^2+ ^when compared to the level of expression in the untreated control plants (Figure 1). Cd^2+ ^and Ni^2+ ^treatment caused a 5.9 and 8.8 fold increase in *BjMTP1 *transcript levels, respectively, compared to untreated control seedlings. Conversely, Zn^2+ ^treated seedlings showed only a minor 1.6-fold increase in *BjMTP1 *mRNA.

### Analysis of the transcriptional competency of the *BjMTP1 *promoter region

**Figure 2:**
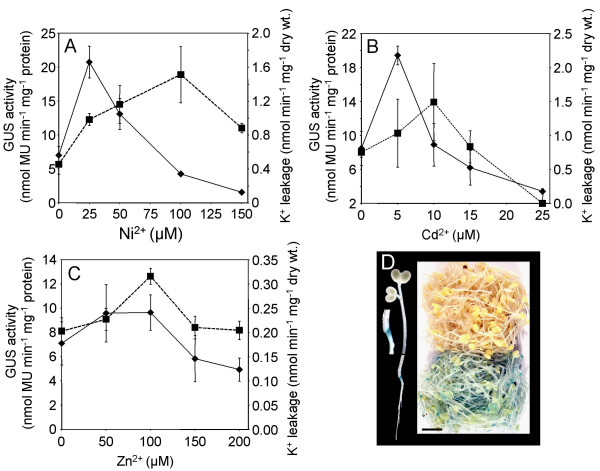
Metal regulated transcriptional activation of *BjMTP1 *by its 983 bp promoter. Five-day-old dark grown seedlings, transformed with *p (1.0) BjMTP1*::*GUSPlus*, were exposed to different concentrations of Ni^2+ ^(**A**), Cd^2+ ^(**B**) or Zn^2+ ^(**C**) and both GUS activity (nmol MU/mg protein/min) and K^+ ^leakage (nmoles K^+^/min/mg dry weight) measured. Each data point represents an average (± SD) of three independent replicate samples. (**D**) Histochemical GUS staining of seedlings exposed to 25 μM Ni^2+ ^for 48 h (bottom) and unexposed seedlings (top). Insert shows the GUS localization in an individual seedling. Scale bar = 1 cm.

A 983 bp DNA fragment, originating upstream of the *BjMTP1 *translational start site, was constructed as a transcriptional fusion with the *GUSPlus *reporter gene [*p(1.0)BjMTP1*::*GUSPlus*]. Five-day-old dark grown seedlings stably transformed with *p(1.0)BjMTP1*::*GUSPlus *were treated with varying concentrations of Ni^2+^, Cd^2+ ^and Zn^2+ ^for 48 h and GUS activity measured (Figure 2). GUS activity was observed to peak at 25 μM Ni^2+^, 5 μM Cd^2+ ^and 75 μM Zn^2+^, with increases in GUS activity of 3.0, 2.3 and 1.3-fold, respectively (Figure 2A–C). The metal concentrations used in this assay spanned a range from moderately to severally toxic, with the highest concentrations of each metal causing complete loss of turger in the seedlings. To assess the level of toxicity at the end of this 48 h assay rates of K^+ ^leakage from the seedlings were measured. All metal treatments caused an increase in K^+ ^leakage peaking at 10 μM Cd^2+^, and 100 μM Ni^2+ ^and Zn^2+^, after which leakage rates dropped and this was associated with a lose of both seedling turger and GUS activity (Figure 2A–C). Histochemical GUS staining of both Cd^2+ ^and Ni^2+ ^treated seedlings demonstrated that the GUS protein product of the *p(1.0)BjMTP1*::*GUSPlus *construct is localized mainly in the roots, showing strong expression at the root-shoot transition zone (Figure 2D). In the untreated transgenic seedlings, there was no observable GUS staining (Figure 2D).

**Figure 3:**
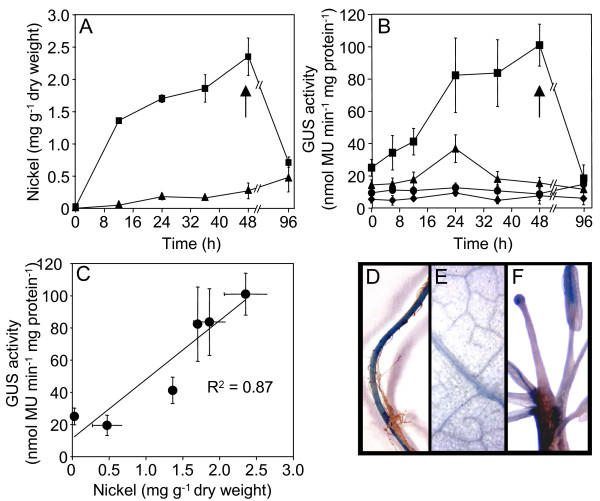
ICP-MS analysis of Ni, GUS enzyme activity and GUS staining of T2 transgenic plants containing *p (1.0) BjMTP1*::*GUSPlus*.(**A**) ICP-MS analysis of Ni in T2 transgenic plants containing *p (1.0) BjMTP1*::*GUSPlus *after exposure to 50 μM Ni^2+ ^in 0.1× Hoagland's nutrient media. After 48 h exposure plants were transferred to 0.1× Hoagland's without Ni^2+ ^(arrow) and Ni^2+ ^accumulation monitored for a further 48 h. Symbols represent roots (squares), and shoots (triangles). (**B**) GUS enzyme activity (nmoles of 4-methyl umbelliferone min^-1 ^mg^-1 ^total protein) measured during the same treatment and time frame as in (**A**). Symbols represent roots (squares), and shoots (triangles) of plants homozygous for the transgene, and roots (circles) and shoots (diamonds) from control plants identified by PCR as null segregants for the transgene. (**C**) GUS enzyme activity and Ni^2+ ^accumulation, from roots exposed to 50 μM Ni^2+ ^for 48 h (data from **A **and **B**). Data represents the average (± SD) of three independent replicate samples for both GUS activity and Ni accumulation. GUS activity visualized by histochemical staining in roots (**D**), leaves (**E**) and floral organs (**F**) from plants exposed to 50 μM Ni^2+ ^for 48 h (roots and floral organs) or 24 h (leaves). **a **stigma, **b **anther.

**Figure 4:**
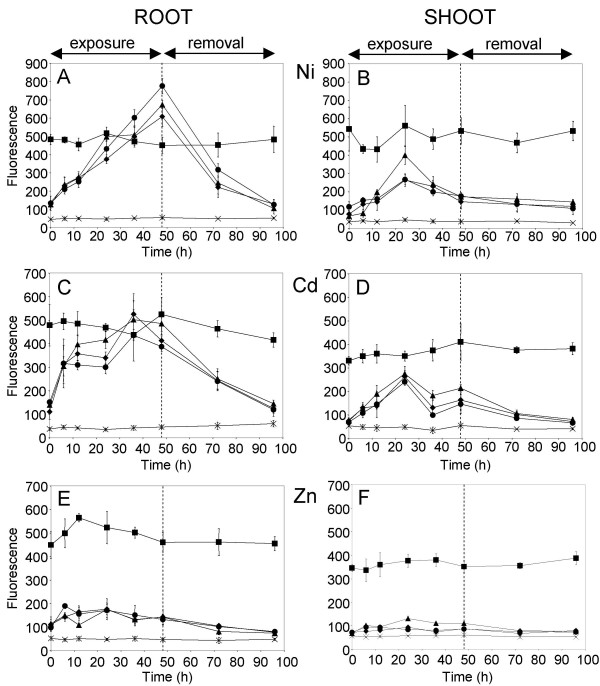
Expression of mRFP driven by varying sized *BjMTP1 *promoter sequences in response to Ni^2+^, Cd^2+ ^and Zn^2+ ^treatment. 48 h metal treated T2 homozygous and null plants were transferred to 0.1× Hoagland's medium with out added metals (arrow) and the accumulation was measured again after 48 h. Metal induced mRFP1 expression and recovery responses were measured by their relative emission fluorescence at 607 nm. Data represents the average (± SD) of three independent replicate samples. Symbols represent null plants (X), *CaMV35S *promoter: mRFP1 (squares), *p (1.0) BjMTP1*::*mRFP1*(triangles), *p (1.6) BjMTP1*mRFP1(diamonds) and *p (1.8) BjMTP1*::*mRFP1*(circles).

Plants stably transformed with *p(1.0)BjMTP1*::*GUSPlus *were also grown in hydroponic culture for four weeks and transferred to medium of the same composition with the addition of 50 μM Ni^2+^. Root and shoot samples were taken over a 96 h time course and both Ni^2+ ^accumulation and GUS activity measured. Ni^2+ ^accumulated in both roots and shoots almost linearly with time, with roots accumulating Ni^2+ ^at approximately 10 times the rate of the shoots (Figure 3A). In root samples, GUS enzyme activity increased linearly as the Ni^2+ ^exposure time increased, with a 3.2 fold increase in GUS activity after 48 h Ni^2+ ^treatment compared to basal expression (0 h), or to untransformed control plants (Figure 3B). In shoots, the overall GUS activity was less than in the roots. Interestingly, in shoots GUS activity only transiently increased after 24 h exposure, compared to untransformed plants, after which GUS activity decreased to levels observed at 0 h exposure (Figure 3B), even though Ni^2+ ^accumulation in shoots continued (Figure 3A). Similar results for both root and shoot expression were also obtained with plants stably transformed with *p(1.0)BjMTP1*::*EYFP *(yellow fluorescent protein) (data not shown), and *p(1.0)BjMTP1*::*mRFP1 *(red fluorescent protein) (Figure 4). A comparison of total tissue Ni^2+ ^accumulation and GUS activity revealed a strong positive correlation between the level of Ni^2+ ^accumulation and GUS activity in roots (Figure 3C). In order to test whether the induction process is reversible, after 48 h Ni^2+ ^treatment plants were transferred to nutrient solution lacking Ni^2+^. After a further 48 h recovery GUS activity in roots was observed to return to that observed at 0 h exposure (Figure 3B). Histochemical GUS analysis of roots from four-week old plants, after 48 h Ni^2+ ^treatment, demonstrated clear GUS expression throughout the main root (Figure 3D). After 48 h Ni^2+ ^treatment GUS expression was not detected in the stem. GUS expression was, however, observed in the vasculature of the leaves (Figure 3E) and in the vascular tissues of the anthers and the stigma (Figure 3F) after 24 hr Ni^2+ ^exposure.

The regulatory competency of the 983 bp, 1,561 bp and 1,786 bp DNA sequences upstream of the *BjMTP1 *translations start site were compared to establish if further metal regulatory elements exist upstream of the 983 bp fragment. The transcriptional activity of these DNA fragments was assessed after construction and transformation of *B. juncea *with transcriptional fusions with the monomeric red fluorescent protein (*mRFP1*) as a reporter gene. Hydroponically grown four-week-old *B. juncea *plants transformed with the *mRFP1 *reporter constructs were exposed to either 5 μM Cd^2+^, 50 μM Ni^2+ ^or 50 μM Zn^2+ ^in aerated 0.1× Hoaglands solution. Root and shoot samples were collected over a 96 h time-course and *mRFP1 *expression quantified using a luminescence spectrometer. As a control for metal related effects on the *in vivo *stability of mRFP1 *pCaMV35S:: mRFP1 *transformed plants were also generated and treated in a similar manner. In roots isolated from Ni^2+ ^treated plants, mRFP1 accumulation increased linearly with time of Ni^2+ ^exposure, and the kinetics of mRFP1 accumulation were equivalent regardless of the size of the promoter construct used to drive *mRFP1 *expression (Figure 4A). All three promoter constructs produced higher mRFP1 accumulation after 48 h Ni^2+ ^exposure than observed with the *CaMV35S *promoter. Plants were removed from the Ni^2+ ^containing medium after 48 h exposure and allowed to recover for a further 48 h. During this recovery period, mRFP1 accumulation decreased linearly with time in a similar manner regardless of the size of the promoter region (Figure 4A). In leaves from Ni^2+ ^exposed plants, all three promoter constructs drove a similar transient increase in mRFP1 accumulation that peaked at 24 h Ni^2+ ^treatment, after which they declined at a similar rate to the response observed for the promoter GUS construct. mRFP1 expression in plants transformed with *pCaMV35S::mRFP1 *was constant at all time points and tissues after Ni^2+ ^exposure.

Similar mRFP1 expression was also obtained when plants were exposed to 5 μM Cd^2+ ^for 48 h and allowed to recover from Cd^2+ ^exposure for a further 48 h. mRFP1 accumulated rapidly in roots after Cd^2+ ^exposure to levels equivalent to expression driven by *pCaMV35S *(Figure 4C). Removal of plants from the Cd^2+ ^containing nutrient solution caused a rapid drop in mRFP1 accumulation, with mRFP1 levels returning to those observed prior to Cd^2+ ^exposure, after 48 h recovery (Figure 4C). Again, all three-promoter sizes gave similar responses. Cd^2+ ^treatment also produced a transient accumulation of mRFP1 in shoots after 24 h exposure (Figure 4D), as observed during Ni^2+ ^exposure (Figure 4B). Unlike Ni^2+ ^and Cd^2+ ^exposure, Zn^2+ ^treatment produced no significant alteration in mRFP1 accumulation in either root or shoot tissue (Figure. 4E &4F). Elemental analysis of the nutrient solutions during the Ni^2+^, Cd^2+ ^and Zn^2+ ^experiments confirmed that concentrations of Ni^2+^, Cd^2+ ^and Zn^2+ ^did not vary significantly in the solution during the course of the experiments (data not shown).

### Response of the 983 bp promoter element to other abiotic stresses

The 983 bp promoter region, which was found to be sufficient for expression in response to Cd^2+ ^and Ni^2+^, was also tested for its ability to activate transcription when seedlings were exposed to other abiotic stresses including cold, heat, NaCl and H_2_O_2_. Five-day-old *B. juncea *seedlings stably transformed with *p (1.0) BjMTP1*::*GUSPlus *were exposed to a cold (4°C) or heat (37°C) shock, and also 100 mM NaCl, or H_2_O_2 _(5 μM, 10 μM, 100 μM 500 μM and 1 mM) after which GUS activity was assayed. There were no significant increases in GUS activity compared to the untreated seedlings for any of the treatments (Table 1).

**Table 1:** Different abiotic stress responses of 983 bp promoter element of *BjMTP1*

Different stress treatments	GUS activity *(nmoles of 4 -methyl umbelliferrone min^-1 ^mg^-1 ^protein)*
Cold shock control	81.01 ± 3.89
Cold shock	87.7 ± 4.61
Heat shock control	84.55 ± 3.13
Heat shock	85.21 ± 6.46
NaCl stress control	99.1± 5.71
100 mM NaCl stress	98.38 ± 8.92
H_2_O_2 _control	91.46± 3.07
50 μM H_2_O_2 _stress	96.16± 0.68

Five-day-old dark grown seedlings stably transformed with *p(1.0)BjMTP1*::*GUSPlus *were incubated at 4°C for 4 h (cold shock), 37° C for 2 h (heat shock),100 mM NaCl (24 h) and 50 μM H_2_O_2 _(24 h) and GUS activity measured. Data represents the average (± SD) of three independent replicate samples.

### Putative regulatory elements in the *BjMTP1 *promoter region

**Figure 5:**
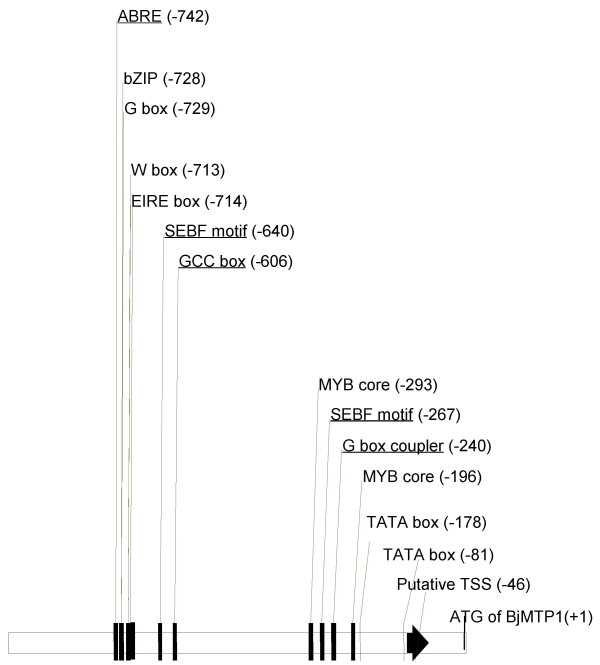
Regulatory motif analysis of a 983 bp sequence upstream of the *BjMTP1 *translational start site was done region using PLACE and PlantCARE databases. Regulatory motif analysis was also done for similar sequences upstream of the transcriptional start sites (TSS) of *AtMTP1 *and *BjMTP1*. Unique elements only identified in *BjMTP1 *are underlined..

A search for putative regulatory DNA elements within the 983 bp promoter region of *BjMTP1 *was performed using the PLACE [18] and PlantCARE databases [19] (Figure 5). DNA regulatory motifs such as the CCAAT boxes, ABRE (Abscisic Acid Response Element), bZIP, G box, and several pathogen responsive elements such as W box, EIRE, SEBF motif, GCC box, G box coupler and MYB core elements were found. However, no known metal regulatory elements such as MREs which are known to mediate Zn^2+ ^and Cd^2+ ^specific transcriptional activation of metallothionine genes in mammals were found [20-22]. Considering that *AtMTP1 *appears not to be regulated by Cd^2+ ^[4] and *BjMTP1 *is (Figure 1, 2, 3, 4), we compared the 983 bp *BjMTP1 *promoter region with a similar region of the *A. thaliana AtMTP1 *sequence upstream of the transcriptional start site (35715 bp to 36698 bp of *A. thaliana *chromosome 2 clone F19D11). Even though both promoter regions had many known regulatory elements in common, namely bZIP, G box, W box, EIRE box and MYB core element, the *BjMTP1 *promoter region also has several unique regulatory elements, namely ABRE and pathogen related SEBF motif, GCC box and G box coupler elements (Figure 5). The core sequence of the metal regulatory element [MRE-TGCRCNC [22]] found in mammals (mouse), is present in the full 1786 bp upstream region of *BjMTP1 *with a single base pair difference (TGCACTG). However, because this motif is not present in the 983 bp promoter region sufficient for Cd^2+ ^and Ni^2+ ^regulation (Figure 2, 3, 4), this MRE is unlikely to play a role in the Cd^2+ ^and Ni^2+ ^transcriptional regulation of *BjMTP1*.

### Localization of *BjMTP1 *expression to root xylem parenchyma

**Figure 6:**
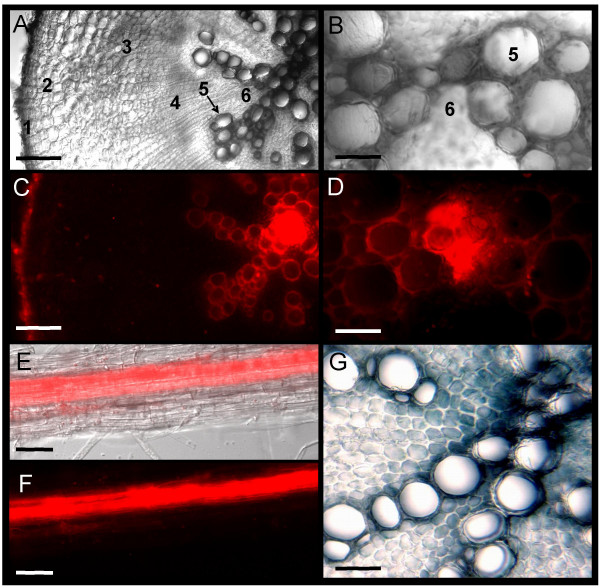
Microscopic analysis of roots from T2 homozygous transgenic plants (*p BjMTP1*::*mRFP1 *and *GUSPlus*) after exposure to 50 μM Ni^2+ ^for 48 h. Images of cross sections taken from the top of the main root under bright field illumination, using diascopic filters at 10× (**A**) and 20× (**B**) magnification, and fluorescent images of the same sections using a Rhodamine – Texas red filter at 10× (**C**) and 20× (**D**) magnification.. Root morphology in (**A**) and (**B**) labeled as follows. (**1**) periderm, (**2**) pericycle, (**3**) secondary phloem, (**4**) cambial zone, (**5**) secondary xylem and (**6**) secondary xylem parenchyma. Lateral root showing an overlay of a bright field and fluorescent image (**E**) and fluorescent image alone (**F**). Image of a root cross section showing GUS activity at 20× magnification. Scale bar = 40 μm for B, D, E, F, G and 120 μm for A and C.

Our results demonstrate that a 983 bp region upstream of the translational start site of *BjMTP1 *is sufficient to drive strong root expression of GUS and mRFP1 in response to Cd^2+ ^or Ni^2+ ^treatment (Figure 2, 3, 4). However, in order to help understand the function of BjMTP1, it is also important to identify which root tissues are expressing *BjMTP1 *during the response to Cd^2+ ^or Ni^2+ ^exposure. Sections from the main root and intact lateral roots of four-week-old *B. juncea *plants expressing *mRFP1 *in response to Ni^2+ ^were analyzed by epifluorescence and light microscope (Figure 6). Sections were taken from the top of the main root as well as 1.5 cm from the root tip, and the morphology of the roots examined (Figure. 6A &6B). The main root has clearly undergone secondary growth. The epidermis found in the primary roots has been replaced in 4-week-old roots by periderm produced by the cork cambium. Within the periderm lies the pericycle, and the cambial zone which gives rise to the secondary phloem and secondary xylem. The secondary xylem parenchyma, produced by vascular cambium, was observed surrounding the secondary xylem vessels. The presence of diarch xylem at the center of the root section is a characteristic of the Brassicaceae family (personal communication G. Myer, South Dakota State University). Sections taken 1.5 cm from the tip of the main root were observed to have similar root architecture to sections taken from the top of the root. After exposure to Ni^2+ ^mRFP1 fluorescence was observed to be specifically localized in the secondary xylem parenchyma tissues that surround the secondary xylem (Figure. 6C &6D). Fluorescence from mRFP1 was also present in the periderm (Figure. 6C) but no significant fluorescence was observed in other root tissues. In the lateral roots, mRFP1 fluorescence was clearly evident in the vascular region (Figure. 6E and 6F). After Ni^2+ ^exposure no fluorescence was observed in roots from plants identified as null segregants for the reporter construct even after longer (6 s) exposures, compared to the shorter (100 ms) exposure for Ni^2+^-treated transgenic plants [see Additional file 2]. Cd^2+ ^treated roots gave an equivalent expression pattern to that of Ni^2+ ^treated plants [see Additional file 2]. In both Ni^2+ ^and Cd^2+ ^treated roots there was no difference in the pattern of mRFP1 accumulation between sections taken from the top of the main root and its tip. Furthermore, mRFP1 expression was localized in the secondary xylem parenchyma tissues when expressed from the 983, 1,561 or 1,786 bp promoter regions. The strong accumulation of mRFP1 observed in the vascular tissue of intact lateral roots (Figure. 6E &6F) is also consistent with the secondary xylem parenchyma localization observed in the root cross-sections. Cross-sections of roots prepared from Ni^2+ ^exposed plants transformed with the *GUSplus *reporter gene also revealed strong GUS expression in the secondary xylem parenchyma surrounding the secondary xylem (Figure. 6G), similar to that observed for mRFP1 expression (Figure. 6C &6D). Secondary xylem parenchyma localization was also observed in plants expressing a *EYFP *reporter driven by the 983 bp promoter region [see Additional file 2].

## Discussion

Here we establish that mRNA levels of *BjMTP1 *in *B*. *juncea *are transcriptionally regulated in response to Cd^2+ ^and Ni^2+^, with maximal expression in the root xylem parenchyma cells. We identify a 983 bp DNA fragment, within the total 1,786 bp DNA sequence upstream of the *BjMTP1 *translational start site, which is sufficient for this transcriptional regulation, which appears specific to stress induced by Cd^2+ ^and Ni^2+^. *BjMTP1 *promoter activity in response to Ni^2+^, Cd^2+ ^and Zn^2+ ^was assessed at concentrations that span a similar range of toxicities from mild to serve. Over these similar ranges of metal-induced stress only Ni^2+ ^and Cd^2+ ^were observed to elicit a transcriptional response from the *BjMTP1 *promoter, with Zn^2+ ^induced stress having no significant effect. Other forms of abiotic stress including cold, heat or NaCl also produced no significant transcriptional response. Transcriptional activation of *BjMTP1 *promoter by Cd^2+ ^and Ni^2+ ^is also not an indirect response to oxidative stress, since this promoter region is not activated by direct exposure to H_2_O_2_.

Our results are consistent with the observation in *A*. *thaliana *that the homologue *AtMTP1 *is also not regulated by Zn^2+ ^[2-4,8]. However, our observation that *BjMTP1 *expression is transcriptionally regulated by Cd^2+ ^and Ni^2+ ^is not consistent with that observed in *A*. thaliana, where *AtMTP1 *mRNA levels are unaffected by Cd^2+ ^[4]. Our results suggests that rather than being a house-keeping gene involved in Zn^2+ ^homeostasis as has been suggested in *A*. thaliana, in *B*. juncea *BjMTP1 *may be involved in the dynamic regulation of Zn homeostasis as part of the plant's response to Cd^2+ ^and Ni^2+ ^induced stress. We emphasize that our data does not suggest that the function of BjMTP1 is to transport Cd^2+ ^or Ni^2+^, for which we have no evidence. However, increased expression of BjMTP1 under Cd^2+ ^or Ni^2+ ^stress may be required to adjust Zn homeostasis as a response to the stress imposed by exposure to Cd^2+ ^or Ni^2+^. This is supported by our observation that expression of *BjMTP1 *promoter is dynamically regulated in response to these metals, rapidly increasing after exposure to Cd^2+ ^and Ni^2+^, and rapidly returning back to basal levels after these metals are removed from the growth medium.

Though expression of the *BjMTP1 *promoter in response to Cd^2+ ^and Ni^2+ ^is highest in roots, expression was also observed in the vascular tissue of leaves, anthers and in the stigma. Expression of *BjMTP1 *in these tissues suggests that the BjMTP1 protein may also be involved in the plants response to Cd^2+ ^and Ni^2+ ^stress in leaves and inflorescences. Though transcriptional activation of the 983 bp promoter region in roots by Cd^2+ ^or Ni^2+ ^occurs nearly in a linear fashion with time of exposure and accumulation, shoot activation appears transient. After 24 h of metal exposure transcription of *BjMTP1 *reaches a maximum and then declines to baseline expression after 48 h. Such transient expression is intriguing considering that over the same time frame Ni^2+ ^accumulates linearly in shoots. The different expression patterns of *BjMTP1 *promoter in roots and shoots imply that this protein plays different roles in the plant's coordinated response to Cd^2+ ^and Ni^2+ ^exposure, though what these roles are remain unclear at this time.

In five-day-old seedlings of *B. juncea *expression of *BjMTP1 *promoter in response to Cd^2+ ^and Ni^2+ ^occurs throughout the seedlings, though is especially localized to the root and shoot-root transition zone. Though the reasons for stronger expression *BjMTP1 *promoter in the shoot-root transition zone are not known, this type of expression pattern has been observed previously for several proteins, including naphthylphthalmic acid (NPA) associated amino peptidases, glutathione *S*-transferase ATGSTF2, the auxin transporter PIN2, and PGP4 an ABC type transporter [23-26].

When compared to *A. thaliana*, the unique elements present in the *BjMTP1 *promoter region, including ABRE, SEBF motif, GCC box and G box coupler elements all have known functions in ABA signaling [27] or pathogen related stress [28-30]. However, none of these promoter elements have been established to play a role in signaling Cd^2+ ^or Ni^2+ ^stress. The elements responsible for regulation of *BjMTP1 *expression in response to Cd^2+ ^and Ni^2+ ^remain to be identified. This study is the first to identify transcriptional regulation and tissue localization of *BjMTP1*. However, further work is needed to understand the functional role of BjMTP1 in *B. juncea's *response to Ni^2+ ^and Cd^2+^.

## Conclusion

Here we conclude that a 983 bp DNA fragment upstream of the *BjMTP1 *translational start site is sufficient for the specific activation by Ni^2+ ^and Cd^2+ ^of *BjMTP1 *expression primarily in roots. Activation of expression by both metals in roots is primarily localized to the xylem parenchyma cells. This study is the first to identify specific Ni^2+ ^and Cd^2+ ^transcriptional regulation and tissue localization of *BjMTP1 *and supports the conclusion that BjMTP1 is involved in the response of *B. juncea *to Ni^2+ ^and Cd^2+ ^exposure.

## Methods

### Plant material

Indian mustard (*Brassica juncea) *seeds (accession no. 426308) were obtained from the North central regional plant introduction station (Ames, IA) *Brassica juncea *seeds (30 seeds) were germinated and grown in 800 mL of continuously aerated distilled water as described in [16]. Seedlings were maintained in the dark at 21°C for 5 days, and the water changed on every third day. *Brassica juncea *plants were also grown hydroponically in 8 L of aerated 0.1× Hoagland's medium [31]., changed weekly, for four weeks with 16 h light (150 μmol m ^-2 ^s^-1^) at 25° C. All transgenic plants used in the study were homozygous for the transgene.

### Cloning of *MTP1 *genomic DNA

Genomic DNA was isolated from 10-day-old *B. juncea*, *B. nigra *and *B. campestris *seedlings using a DNeasy plant mini kit (Qiagen, Valencia, CA). Taking advantage of the fact that *AtMTP1 *contains no introns, we isolated full length *BjMTP1 *cDNA by PCR using 5'-ATGGAGTCTTCAAGTCCCCA- 3'(forward primer) and 5'-TAGAGCGCTCGATTTGTAT-3' (reverse primer) primers based on the *AtMTP1 *sequences. After obtaining genomic sequences 5' of *BjMTP1 *by genome walking (see below) *BjMTP1, BnMTP1 *and *BcMTP1 *genomic DNAs were reamplified using forward primer 5'- ATGGCGTATTCAAGCCCCCAACG- 3' and reverse primer 5'- GCTCTAGAGCGCTCGATTTGTATGG -3' designed based on the sequence of *BjMTP1*. Conditions used in all the PCR reactions are as follows: initial denaturation 94°C followed by 30 cycles of 94° C 30 sec, 55° C 40 sec, 72° C 1 min and 30 sec and final extension at 72° C for 10 min. PCR products was cloned into the pGEM T-Easy vector system and sequenced using Big dye terminator v 3.0 method (Applied Biosystems Foster city, CA) with universal M13 primers.

### Analysis of *BjMTP1 *mRNA expression in five day old seedlings

For metal treatment five-day-old dark grown seedlings were transferred to distilled water containing 25 μM Ni^2+^, 75 μM Zn^2+ ^or 5 μM Cd^2+ ^and incubated for a further 48 h in the dark with aeration. After metal treatment seedlings were washed in distilled water before proceeding to further analysis. Three independent replicate samples were used for all analyses. Metal concentrations were chosen based on the concentrations that produced the maximal increase in GUS activity in transgenic *B. juncea *containing a *BjMTP1 *promoter::GUS construct (see below).

Total RNA from metal exposed seedlings was isolated using the RNeasy mini kit (Qiagen, Valencia, CA) using the DNase digestion step to reduce DNA contamination. cDNA was reverse transcribed using 4 μg of total RNA and 200 U Superscript III reverse transcriptase (Invitrogen, CA) primed with 100 ng of random hexamers (Invitrogen life technologies, Carbsbad, CA), and the cDNA diluted to 2.6 ngμL^-1^. Real time quantitative PCR was done as previously described [32]. BjACTIN2 was used as a normalization control for the relative quantification of transcript levels. Since the B. juncea ACTIN2 gene sequence was not available A. thaliana ACTIN2 primers (forward primer-5'- AAGATCTGGCATCACACTTTC- 3', reverse primer-5'-TAGTCAACAGCAACAAAGGAG- 3') were used to amplify a 529 bp homolog from B. juncea. For qRT-PCR primers were designed to generate ~100 bp products using Primer Express v. 2.0 software (Applied Biosystems, Foster City, CA, USA). Primers used for qRT-PCR were as follows: BjACTIN2 forward primer-5'-GAGGATGGCATGTGGAAGAGA- 3', reverse primer- 5' -GTGCTGGATTCTGGTGATGGT- 3', BjMTP1 forward primer- 5'-TGCGGCTTCTCAGATCTCAA- 3' and reverse primer-5'-TGCGCATGGAGGCATTG- 3'. Quantitative RT- PCR was performed on an ABI Prism 7000 Sequence Detection System (Applied Biosystems Foster City, CA, USA), following the manufacturers recommendations, and optimized primer concentrations were selected based on denaturing curve analysis and the fewest cycles needed to cross the critical threshold (Ct). Four reactions were done per biological sample and three independent replicate samples per treatment were used. SYBR Green PCR Master Mix (Applied Biosystems) was used to detect cDNA amplification. Data was analyzed using the SDS software (Applied Biosystems version 1.0), following the method of Livak and Schmittgen [33]. Ct values were determined based on efficiency of amplification. The mean Ct values were normalized against the corresponding *BjACTIN2 *Ct values and calculated as (Ct _*BjMTP*1_- Ct _*BjACTIN*2_). The relative expression of *BjMTP1 *was calculated as fold induction from untreated seedlings using the 2^-ΔΔCt ^method (ΔΔCt = (Ct_BjMTP1_-Ct_BjACTIN2_) metal treated – (Ct_BjMTP1 _- Ct_BJACTIN2_) untreated [33]). The data is presented as fold change in *BjMTP1 *gene expression (normalized to *BjACTIN2*) relative to the untreated control seedlings. For untreated control samples 2^-ΔΔCt ^is one since ΔΔCt is zero. The final standard error was estimated by evaluating the 2^-ΔΔCt ^term using ΔΔCt plus standard deviation and ΔΔCt minus the standard deviation [33].

### *BjMTP1 *promoter::report gene construction

For the isolation of the 5' sequence upstream of the *BjMTP1 *translational start site we used a Universal Genome Walker kit (Clontech, Mountain View, CA) following the manufacturer's protocol. First round of DNA fragment amplification used *BjMTP1 *gene specific primers, GSP1 5'-GAAGAAAGCACGACAAGTCTGGCAAGTTTA-3' and GSP2 -5'- CGTGCTTTCTTCAACGGCTTTGCTGC- 3' (nested), located (+) 49 to (+) 78 bp and (+) 3 to (+) 31 bp relative to the translational start site. For second round amplification primers were 5'-CTGCTAGCTTCTTCCTGCAATCATAT-3' and 5'-ACCTGTAGTAATCAACCTCAGTTACC-3 (nested) located (-) 1302 to (-) 1328 bp and (-)1337 to (-) 1357 bp relative to translational start site. PCR amplified products were cloned into either pGEM T-Easy vector system (Promega Corporation, Madison, WI) or PCR-XL-TOPO vector (Invitrogen Life Technologies, Carbsbad, CA). Cloned constructs were transformed into chemically competent *E. coli *Top10 F^- ^cells (Invitrogen Life Technologies, Carbsbad, CA). Positive clones were sequenced using Big dye terminator v 3.0 method (Applied Biosystems Foster city, CA) with universal M13 primers. DNA sequences were analyzed by BlastN [34] and known regulatory elements identified using PLACE [18] and PlantCARE [19]. Putative eukaryotic promoter analysis was done using BGDP, a neural network eukaryotic promoter prediction program [35]. *BjMTP1 *promoter sequence has been submitted to Genbank (#EF128447).

The *GUSPlus *gene was PCR amplified from pCambia 1305.1 binary vector using high fidelity Platinum TAQ DNA polymerase (Invitrogen life technologies, Carlsbad, CA) and gene specific primers complementary to the *GUSPlus *gene. The sense and antisense primers contained engineered EcoRI and BamHI sites, respectively. The GUSPlus primers used were: GUSPlus-BamHI-5'-CGGGATCCCATGGTAGATCTGAGGGTAAATTTCTAGT- 3', GUSPlus EcoRI -5'-CGGAATTCTCACACGTGATGGTGATGGTGATGGCTAGC- 3'. PCR products were cloned into pGEM T-Easy vector. The cloned PCR products were chemically transformed into *E. coli *DH5α, positive clones digested with BamHI and EcoRI and the released cDNAs cloned into the *CaMV35S *cassette-pUC19 [36] using the same restriction enzyme sites. The insertion of the cDNA product in the correct orientation was confirmed by PCR using promoter specific and 35S PolyA tail specific primers (5'-ATAAGAATGCGGCGATATCGATATCGATCTGGATTTTAGTA-3'), and further confirmed by sequencing. Using unique EcoRV sites the entire promoter cassettes were cloned into pGreen 0229 binary vector containing the *bar *gene as a selectable marker. mRFP1 [37] was directly isolated from the pRSET B vector and cloned into *CaMV35S *cassette using BamHI-EcoRI sites.

Based on the previously identified sequence information, different sizes of *BjMTP1 *promoter regions were PCR amplified using high fidelity platinum Taq DNA polymerase with 5' PstI- 3' BamHI promoter specific primers. The primers used were Bj 983 bp 5' PstI 5'-AACTGCAGAGTTTCCATTTTTGTTTTCGTGCTAAATAA-3', Bj1561 bp 5' PstI-5'-AACTGCAGTGCACTGATGAAGTTCCGGATGAAGAGGAA-3', Bj 1786 bp 5' PstI-5'-AACTGCAGACAGACAAAACCAGTTTCTTCAGTCCGGGA-3' and Bj rev BamHI-5'-CGGGATCCTCTGAAAAGAAAAAAATCAGAGAAAGTTCA-3'. The PCR amplified *BjMTP1 *promoter regions were cloned into PCR-XL-TOPO vector. Positive clones containing PCR-XL-TOPO: *BjMTP1 *promoter regions were digested with PstI and BamHI and the released DNA cloned into pGreen 0229 containing specific reporter gene cassettes outlined above, after removing the *CaMV35S *promoter from the vector by PstI-BamHI digestion. All the cloned promoter regions (983 bp, 1,561 bp and 1,786 bp) contained the respective 5' UTR of the *BjMTP1 *gene and all the constructs contained only the start codon from their respective reporter genes. The ligated constructs were transformed into *E. coli *DH5α. Positive clones were identified by PCR using a combination of promoter specific and reporter gene specific primers and further confirmed by sequencing. All the pGreen constructs were mixed with pSoup plasmid DNA and transformed into electro competent disarmed *Agrobacterium tumefaciens *GV3101. Positive clones were selected by colony PCR.

### Plant transformation and regeneration

Transgenic *B. juncea *plants were obtained by tissue culture based on a previously established protocol (personal communication Thomas Leustek, Rutgers University). Briefly, petioles were excised from cotyledons of five-day-old *in vitro *grown seedlings on agar solidified half strength MS medium [38] and cultured in MS medium with 3 % (w/v) sucrose, 2.5 g L^-1 ^Gelrite, 2 mg L ^-1 ^6-benzylaminopurine, and 0.1 mg L^-1 ^naphthalene acetic acid under a 16 hour photoperiod (100 μmol m ^-2 ^s ^-1^) at 25°C for 2 days. Petioles were transformed with *A. tumefaciens *GV3101 harboring pGreen 0229 containing *BjMTP1 *promoter reporter gene constructs.*Agrobacterium tumefaciens *strain GV3101 carrying the pGreen vectors were grown for 48 h in 30 mL liquid YEP medium containing 1 g L^-1 ^yeast extract, 5 g L^-1 ^beef extract, 5 g L^-1 ^bacto-peptone, 5 g L^-1 ^sucrose, 0.5 g L^-1 ^MgSO_4_.7H_2_O and 100 mg L^-1 ^kanamycin in a 28°C shaker at 250 rpm until the culture reached an OD_600 _of 0.7. Bacteria were harvested by centrifugation and the bacterial pellet resuspended in 30 mL MS liquid medium. Petioles were incubated with the *A. tumefaciens *suspension for 20 min, blotted dry using sterile filter paper and transferred to freshly prepared tobacco (BY2 cell line) feeder layer plates. After 48 h incubation in the dark, petioles were rinsed in medium containing MS salt, 3% (w/v) sucrose, and 500 mg L^-1 ^carbencillin for 40 min. The petioles were cultured on MS medium containing 2 mg L^-1 ^TDZ and 0.1 mg L^-1 ^IAA with 500 mg L^-1 ^carbenicillin for three to four weeks under 16 h photoperiod (100 μmol m ^-2 ^s ^-1^). Roots were induced from regenerated shoots in solid MS medium containing 2 mg L^-1 ^indole-3- butyric acid. Plantlets were later transferred to soil for further growth. Putative transformants were selected based on their ability to grow in regeneration medium containing 3 mg L^-1 ^glufosinate ammonium (active ingredient of Basta). Transformants were also analyzed for the presence of the introduced DNA constructs using PCR. T2 and T3 homozygous lines were selected by spraying BASTA (8 mg L^-1^) on 15-day old plants with fully expanded first leaves (total of 4 applications on every third day), and further confirmed by PCR.

### Analysis of *BjMTP1 *promoter activity

For analysis of metal regulated promoter activity five-day-old dark grown seedlings were treated with various concentrations of Ni^2+^, Cd^2+ ^and Zn^2+ ^in distilled water for 48 h in the dark. For heat shock similar seedlings were incubated at 37°C for 2 h as described in [39]. Cold shock was given at 4°C for 4 h following the method of [40]. For salt treatment seedlings were treated with 100 mM NaCl as described in [41] for 24 h in the dark with aeration. Seedlings were also exposed to H_2_O_2 _at 5 μM, 10 μM, 50 μM, 100 μM, 500 μM and 1 mM H_2_O_2 _for 24 h, changing solutions every 12 h. For metal treatment in mature *B. juncea *four-week-old hydroponically grown plants were transferred to aerated 0.1 × Hoagland's solution containing 50 μM Ni^2+^, 50 μM Zn^2+ ^or 5 μM Cd^2+^. To maintain a constant Cd^2+^concentration throughout the experiment the hydroponic media was replaced at 12 h intervals. Because of the higher initial concentration of Ni^2+ ^and Zn^2+ ^their concentrations were found to not significantly change during the experiment, as analyzed by ICP-MS (data not shown). For recovery experiments plants were transferred to aerated 0.1 × Hoagland's solution lacking either Ni^2+^, Cd^2+ ^or elevated Zn^2+^. Root and shoot samples were also analyzed for Ni^2+ ^accumulation by ICP-MS. Three independent replicate samples were used for each analysis. For total GUS activity tissue samples were homogenized in liquid nitrogen and suspended in 200 μL of extraction buffer (50 mM NaHPO_4 _pH 7.0, 2 mM DTT, 10 mM Na_2 _EDTA, 0.1 % (w/v) sodium lauryl sarcosine and 0.1 % (v/v) Triton X- 100; [42]) and the soluble protein fraction collected after centrifugation at 10,000 × g. Quantitative fluorometric analysis of GUS enzymatic activity was carried out according to [42]. Fluorescence was measured using a luminescence spectrometer (PerkinElmer model # LS 55, Life and Analytical Sciences Boston, MA) after 60 min incubation with the GUS substrate. Each assay was performed using three independent replicate samples. Total protein content was determined using a BCA protein assay kit (Pierce Biotechnology, Rockford, IL). GUS activity data is expressed as nmoles of 4-methylumbelliferone(MU)min^-1 ^mg^-1 ^of extracted protein. Histochemical GUS analysis of *B. juncea *tissues was performed based on a standard protocol [43]. GUS localization was analyzed using an Olympus Vanox light microscope (Olympus America Inc. Melville, NY). For photography a SPOT-RT digital camera (Diagnostic instruments Sterling Heights, MI) attached to the microscope was used. For quantitative fluorometric assay of mRFP1 in tissue samples 100 mg fresh weight of tissue was homogenized in liquid nitrogen and suspended in 200 μL of the PBS buffer (10 mM potassium phosphate buffer, 140 mM NaCl, pH 7.4) containing 1 mM DTT, and the soluble protein fraction collected after centrifugation at 10,000 × g. The relative mRFP1 emission (607 nm) in the samples was measured using a luminescence spectrometer (model # LS 55 PerkinElmer Life and Analytical Sciences Boston, MA) after excitation at 584 nm. Each assay was performed using three independent replicate samples. For epifluorescence microscopy tissues were immediately incubated in PBS buffer and hand sections prepared for localization of mRFP1 expression. Epifluorescence was observed using Nikon E 800 compound microscope equipped with Rhodamine – Texas red (exciter λ 560 ± 20, dichroic λ Q 585 LP and emitter λ 610 LP) filter. For photography SPOT-RT digital camera attached to the microscope was used.

### Metal Toxicity Measurements

Metal toxicity measurements were done using K^+ ^leakage in five-day-old seedlings containing *p(1.0)BjMTP1*::*GUSPlus*, following a method adapted from [44]. After metal treatment seedlings (n = 5) were removed and placed in 10 mL of distilled water for 60 min, the solution filtered through a 0.2 μm filter and K^+ ^measured using ICP-MS. Untreated seedlings were used as a control. Seedlings used for K^+ ^efflux were dried at 68°C for 48 h and K^+ ^leakage expressed on a dry weigh basis.

### Inductively Coupled Plasma – Mass Spectroscopy

Plant tissue sample were washed once in 18 MΩ, dried overnight at 90°C weighed and digested in concentrated HNO_3 _acid (OmniTrace, EM) at 110°C for 4 h. Ni, Cd and Zn were quantified in the samples using an ICP-MS (Elan DRCe, PerkinElmer) following our published methods [45]. K concentration in K^+^-leakage assays were measured directly in the assay solution using ICP-MS (Elan DRCe, PerkinElmer).

## Authors' contributions

DES conceived the work and guided the experiments; BM was primarily responsible for carrying them out with BY contributing to the cloning. All authors have read and approved the final manuscript

## Supplementary Material

Additional file 1 Dendrogram of plant MTP1 protein-coding DNA sequencealignments using Neighbor-Joining. A phylogenetic analysis showing the relationships between the plant MTP1 protein-coding DNA sequences. Click here for file

Additional file 2 Tissue localization of MTP1 expression in *B. juncea*. A microscopic analysis of root cross-sections and lateral roots from 4-week-old *B. juncea *transformed with *p BjMTP1*::*mRFP1 *or *p BjMTP1*::*EYFP *after exposure to 5 μM Cd^2+ ^or 50 μM Ni^2+ ^for 48 h. Click here for file
