# Supplementary material for: Transcriptional activation and localization of expression of Brassica juncea putative metal transport protein BjMTP1
Source: BMC Plant Biol. 2007 Jun 18;7:32. doi: 10.1186/1471-2229-7-32 (PMC1906783; doi:10.1186/1471-2229-7-32)
Supplement: Additional file 1 — Dendrogram of plant MTP1 protein-coding DNA sequencealignments using Neighbor-Joining. A phylogenetic analysis showing the relationships between the plant MTP1 protein-coding DNA sequences. [file 1471-2229-7-32-S1.ppt]

## Slide 1
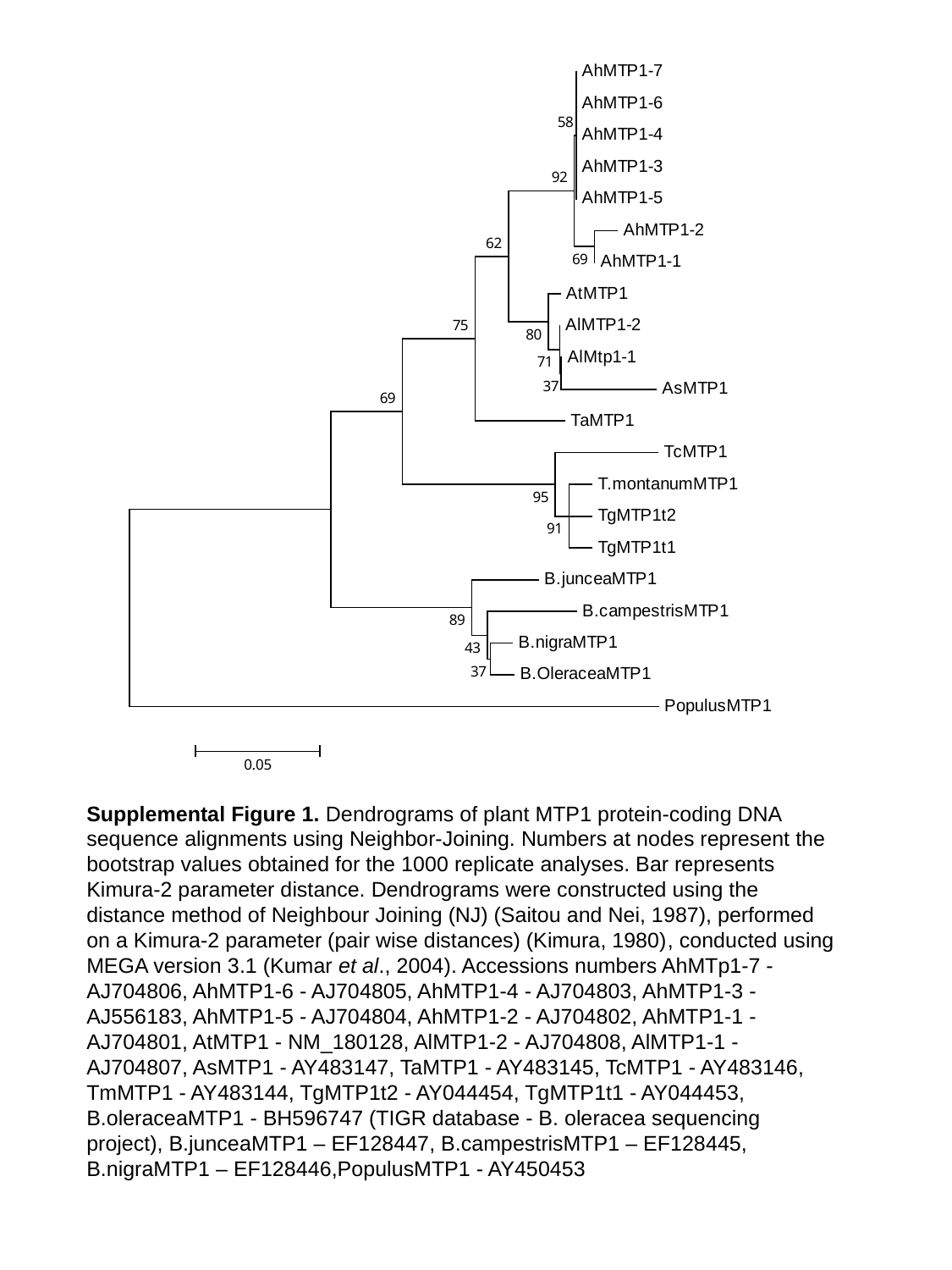

Supplemental Figure 1. Dendrograms of plant MTP1 protein-coding DNA sequence alignments using Neighbor-Joining. Numbers at nodes represent the bootstrap values obtained for the 1000 replicate analyses. Bar represents Kimura-2 parameter distance. Dendrograms were constructed using the distance method of Neighbour Joining (NJ) (Saitou and Nei, 1987), performed on a Kimura-2 parameter (pair wise distances) (Kimura, 1980), conducted using MEGA version 3.1 (Kumar et al., 2004). Accessions numbers AhMTp1-7 - AJ704806, AhMTP1-6 - AJ704805, AhMTP1-4 - AJ704803, AhMTP1-3 - AJ556183, AhMTP1-5 - AJ704804, AhMTP1-2 - AJ704802, AhMTP1-1 - AJ704801, AtMTP1 - NM_180128, AlMTP1-2 - AJ704808, AlMTP1-1 - AJ704807, AsMTP1 - AY483147, TaMTP1 - AY483145, TcMTP1 - AY483146, TmMTP1 - AY483144, TgMTP1t2 - AY044454, TgMTP1t1 - AY044453, B.oleraceaMTP1 - BH596747 (TIGR database - B. oleracea sequencing project), B.junceaMTP1 – EF128447, B.campestrisMTP1 – EF128445, B.nigraMTP1 – EF128446,PopulusMTP1 - AY450453
