# Supplementary material for: Transcriptional activation and localization of expression of Brassica juncea putative metal transport protein BjMTP1
Source: BMC Plant Biol. 2007 Jun 18;7:32. doi: 10.1186/1471-2229-7-32 (PMC1906783; doi:10.1186/1471-2229-7-32)
Supplement: Additional file 2 — Tissue localization of MTP1 expression in B. juncea. A microscopic analysis of root cross-sections and lateral roots from 4-week-old B. juncea transformed with p BjMTP1::mRFP1 or p BjMTP1::EYFP after exposure to 5 μM Cd2+ or 50 μM Ni2+ for 48 h. [file 1471-2229-7-32-S2.ppt]

## Slide 1
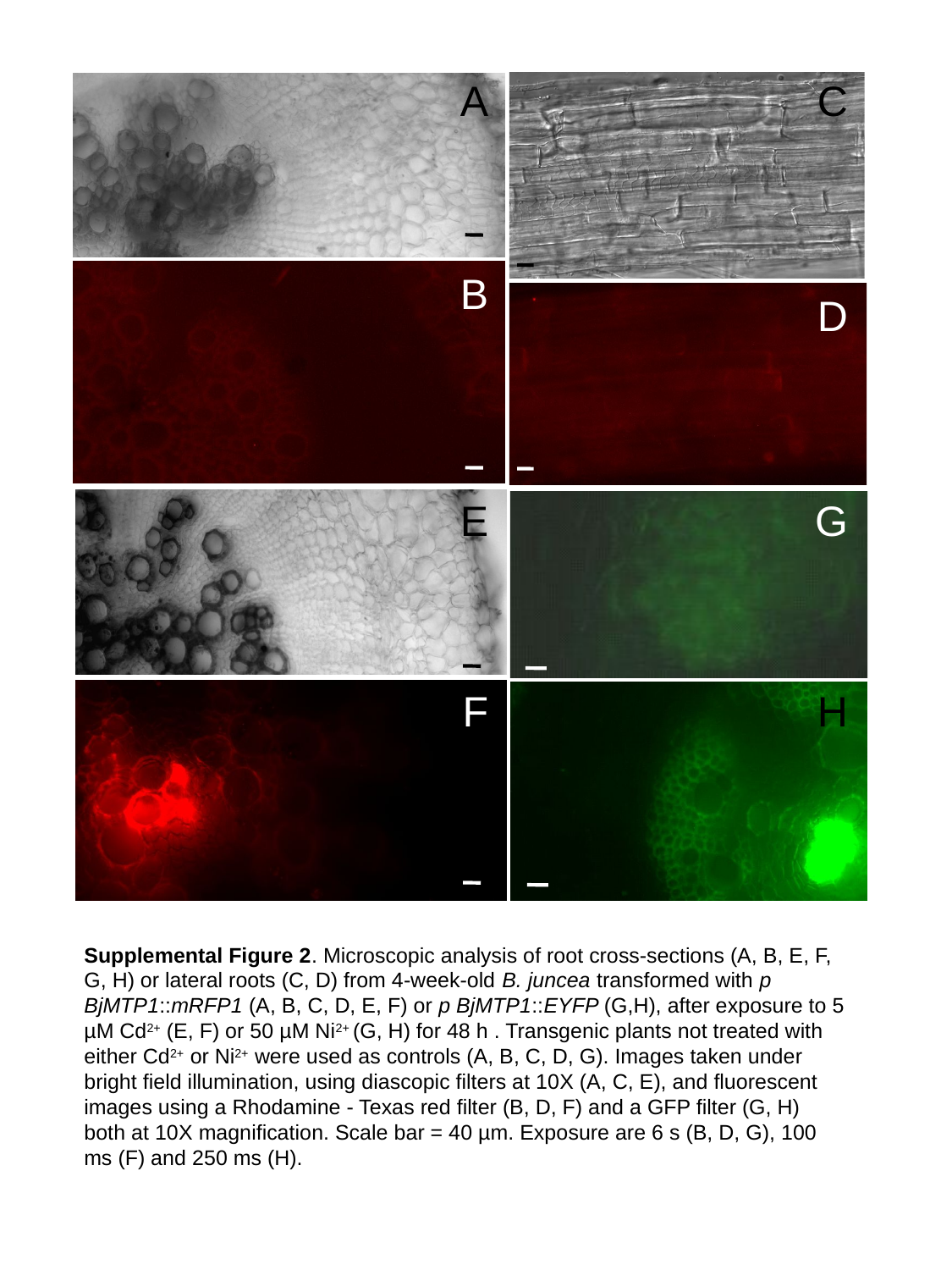

A
C
B
D
E
G
F
H
Supplemental Figure 2. Microscopic analysis of root cross-sections (A, B, E, F, G, H) or lateral roots (C, D) from 4-week-old B. juncea transformed with p BjMTP1::mRFP1 (A, B, C, D, E, F) or p BjMTP1::EYFP (G,H), after exposure to 5 µM Cd2+ (E, F) or 50 µM Ni2+ (G, H) for 48 h . Transgenic plants not treated with either Cd2+ or Ni2+ were used as controls (A, B, C, D, G). Images taken under bright field illumination, using diascopic filters at 10X (A, C, E), and fluorescent images using a Rhodamine - Texas red filter (B, D, F) and a GFP filter (G, H) both at 10X magnification. Scale bar = 40 µm. Exposure are 6 s (B, D, G), 100 ms (F) and 250 ms (H).
